# Patient and Caregiver Perspectives on Their Experiences With Crohn’s Perianal Fistulas

**DOI:** 10.1093/crocol/otad081

**Published:** 2024-01-06

**Authors:** Sylvie Stacy, Emily Belcher, Pradeep P Nazarey, Susan E Cazzetta, Gregory D Salinas

**Affiliations:** CE Outcomes, LLC, Birmingham, AL, USA; CE Outcomes, LLC, Birmingham, AL, USA; Takeda Pharmaceuticals USA, Inc., Lexington, MA, USA; Takeda Pharmaceuticals USA, Inc., Lexington, MA, USA; CE Outcomes, LLC, Birmingham, AL, USA

**Keywords:** Crohn’s disease, perianal fistula, survey, attitudes, challenges

## Abstract

**Background:**

Little is known about patients’ perception of care and management of Crohn’s perianal fistulas (CPF). This study was conducted to understand US patient and caregiver attitudes and challenges to CPF care.

**Methods:**

Patients with CPF and caregivers of patients with CPF completed a 36-question survey about their perceptions and challenges regarding the diagnosis, treatment, and overall management of CPF. Patients/caregivers were recruited via online Crohn’s and fistula support group websites and forums and via their gastroenterologists (GEs) and surgeons from October 2020 through January 2021.

**Results:**

The survey was completed by 96 patients and 54 caregivers. Respondents reported over 60% and 14%–23% of CPF were diagnosed and treated by a GE or surgeon, respectively. Nearly all patients/caregivers wanted to be involved in treatment decision-making with their physicians (81%). While the majority of patients/caregivers were satisfied with their quality of care (65%) and access to care (67%), racial disparities exist and there is room for improvement. A smaller proportion of non-White versus White patients/caregivers reported satisfaction with care quality (39% vs 72%, respectively) and access to care (57% vs 69%, respectively). Half of non-White patient/caregivers (50%) versus 69% of White patient/caregivers knew where to access CPF information. Most patients/caregivers (69%) stated that they would benefit from more information on managing day-to-day CPF symptoms. Significant barriers perceived by patients/caregivers to receiving optimal CPF care included lack of effective treatments (69%) and lack of access to specialist care (68%).

**Conclusions:**

Improvements in multidisciplinary CPF care are required to optimize treatment.

## Graphical Abstract

**Graphical Abstract:**
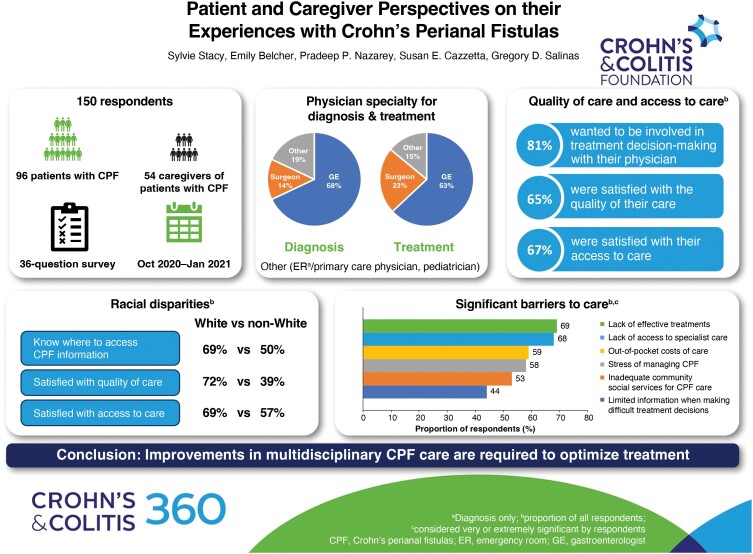

**Key Messages**

**What is already known?**

Management of Crohn’s perianal fistulas (CPF) is challenging and requires a multidisciplinary approach.

**What is new here?**

This study provides an understanding of patient/caregiver attitudes and challenges related to CPF care, including observed racial disparities.

**How can this study help patient care?**

Awareness of patient/caregiver perspectives on their CPF care can be used to improve the quality of care of CPF, including educational initiatives for patient/caregivers and healthcare professionals, particularly with regard to incorporating the patients’ perspective into decision-making, integrating quality-of-life aspects, and coordination of care.

## Introduction

Perianal fistulas are one of the most common complications of Crohn’s disease (CD), occurring in 14%–38% of patients.^1–4^ They are associated with high disease burden, impaired quality of life, and increased use of healthcare services compared with patients with CD without perianal fistulas.^5–8^ Treatment usually requires a combination of medical and surgical therapies.^1,6^

The effective management of Crohn’s perianal fistulas (CPF) is challenging due to the complexity of this complication. Several US clinical practice guidelines have been developed; however, the approach taken by each guideline differs and the recommendations also vary.^9–11^ Despite the available guidelines, an unmet need remains for medical and/or surgical treatments with long-term success rates for CPF.^6^

Evidence-based practice does not necessarily reflect the actual experiences of patients. For example, there is often a mismatch between how surgeons perceive the importance of issues to patients and the patient’s own preferences around perianal fistula surgery.^12^ Previous research relating to inflammatory bowel disease has provided an initial understanding of patients’ perceptions of treatment and experiences with CD.^13–16^ However, little is known regarding patient perceptions of care and management as it relates to CPF as a manifestation of their disease. Research has revealed that patients experience a burden of symptoms, a burden of treatment, and impacts on physical, emotional, and social aspects of well-being, and identified a need for capturing patient experience beyond symptoms.^17^

To supplement a study focusing specifically on physician management of patients with CPF, a survey was conducted to understand patient and caregiver attitudes and challenges related to their CPF care. It was anticipated that this would provide added perspective for the continuing educational needs of physicians and would assist with enhanced design of patient and caregiver initiatives regarding CPF.

## Materials and Methods

### Survey Design and Development

A survey was developed to provide insight into US patient and caregiver perceptions and challenges regarding the diagnosis, treatment, and overall management of CPF. In total, 36 questions were categorized into several broad areas, including demographics and patient characteristics, course of CPF onset and diagnosis, treatment concerns, goals of care, barriers to care, perceptions of care providers, and educational needs (Supplementary Information 1). Patients and caregivers aged 21 years or older were eligible to provide a survey response (caregivers responded for patients aged < 21 years). They were also eligible if they were US residents and were able to read and speak English and understood the instructions and questions provided in the survey. Patients were eligible if they had CPF (self-reported), and caregivers were eligible if they were a current caregiver of a patient with CPF. Respondents were considered caregivers if they were a parent or family caregiver (non-parent) of a patient with CPF. Patients could have more than one caregiver (eg, both parents) complete the survey on their behalf. No relationships existed between the patient and caregiver respondents included in the survey. The protocol for this study was determined to be exempt from review by the Western Institutional Review Board (Puyallup, WA) under 45 CFR § 46.104(d)(2).

Question types included multiple choice, Likert scale, ranking, and free text response. Respondents were asked to supplement their responses with written information for certain questions and to provide overall comments at the end of the survey.

### Survey Distribution and Data Collection

Survey respondents were recruited from October 2020 through January 2021 via posts on online CD and fistula support group websites and forums. Additionally, 133 US gastroenterologists (GEs) and surgeons who “opted-in” to receive invitations and recently participated in similar educational research studies were emailed invitations to distribute to their patients with CPF across the United States.

The survey was expected to take approximately 15 minutes to complete. A monetary incentive (equivalent to US$15) was offered to patients and caregivers for their participation. Physicians were not incentivized for distributing invitations.

### Data Analysis

Descriptive statistics were conducted on key items of the survey, using χ^2^ analysis for categorical variables and *t-*tests for continuous variables. Subanalyses were conducted to compare responses between patients and caregivers, and among respondents of different demographics and other variables related to CPF management. Statistical analysis was conducted using SPSS Statistics 27^®^ (IBM, Armonk, NY). Values were considered significant when *P* < .05.

## Results

### Patient Characteristics

The survey was completed by 150 individuals, comprising 96 patients and 54 caregivers of patients with CPF. Details of their characteristics and demographics are shown in Table 1. The average age of all patients (*n* = 150) was 34 years (range 3–89 years). The mean (range) age of patients who directly responded to survey was 33 (21–72) years, and the mean (range) age of patients for whom caregivers responded on their behalf was 37 (3–89) years. Of the caregiver respondents, all were parents or other family members of patients and 19% cared for a pediatric patient. Most respondents had health insurance, with more patients reporting coverage by private insurance (67%), while more caregivers reported coverage by Medicare/Medicaid (54%). Only 5% were uninsured. Insurance coverage varied by race/ethnicity, with non-White respondents mainly reporting coverage by Medicare/Medicaid (68% vs 32% of White respondents) and White respondents mainly reporting coverage by private insurance (64% vs 25% of non-White respondents). One-third of all respondents reported receiving support to help to pay for CPF care or medication (50% for non-White vs 29% for White respondents).

**Table 1.** Patient demographics and clinical characteristics as reported by patients and caregivers.

	Patients	Caregivers	Total
	*n* = 96	*n* = 54	*n* = 150
Patient age, years, mean (range)	33 (21–72)	37 (3–89)^a^	34 (3–89)
Caregiver age, years, mean (range)		42 (22–73)	
Gender^b^, *n* (%)			
Male	60 (63%)	24 (44%)	84 (56%)
Female	36 (38%)	29 (54%)	65 (43%)
Nonbinary, self-described	0 (0%)	1 (2%)	1 (1%)
Race/ethnicity^b^, *n* (%)			
White	83 (86%)	37 (69%)	120 (80%)
Non-White	12 (13%)	16 (30%)	28 (19%)
American Indian/Alaska native	2 (2%)	5 (9%)	7 (5%)
Black/African American	4 (4%)	2 (4%)	6 (4%)
Hispanic/Latino	5 (5%)	7 (13%)	12 (8%)
Other	1 (1%)	2 (4%)	3 (2%)
Undisclosed	1 (1%)	1 (2%)	2 (1%)
Received support to help to pay for CPF care or medication? *n* (%)			
Yes	32 (33%)	17 (31%)	49 (33%)
No	62 (65%)	34 (63%)	96 (64%)
Unsure	2 (2%)	3 (6%)	5 (3%)
Patient health insurance status, *n* (%)			
Yes, private	64 (67%)	21 (39%)	85 (57%)
Yes, Medicare/Medicaid	29 (30%)	29 (54%)	58 (39%)
No	3 (3%)	4 (7%)	7 (5%)
Annual household income, *n* (%)			
Under $50 000	19 (20%)	20 (37%)	39 (26%)
$50 000–$90 000	57 (59%)	14 (26%)	71 (47%)
Over $90 000	20 (21%)	20 (37%)	40 (27%)
Years since CPF onset, mean (range)	4.9 (0–48)	3.6 (0–20)	4.5 (0–48)
Years since CPF diagnosis, mean (range)	3.7 (0–35)	3.0 (0–20)	3.5 (0–35)
Diagnosed with “complex” fistula			
Yes	73 (76%)	32 (59%)	105 (70%)
No	16 (17%)	16 (30%)	32 (21%)
Unsure	7 (7%)	6 (11%)	13 (9%)

^a^Mean (range) age of patients for whom caregivers responded on their behalf in the survey.

^b^Refers to the gender or race/ethnicity of the patient respondent or caregiver respondent.

CPF, Crohn’s perianal fistulas.

### Disease Burden

Patients and caregivers reported on the burden of CPF. Most respondents indicated that CPF increases stress (66%), disrupts their career (62%), increases their financial burden (60%), and creates an emotional burden (64%). For non-White respondents versus White respondents, a higher proportion reported financial burden (64% vs 59%, respectively) and emotional burden (68% vs 63%, respectively). Overall, the majority of respondents felt comfortable discussing CPF with their friends or family members (57%) and reported feeling a sense of community among people/families with CPF (58%). However, for non-White respondents versus White respondents, a lower proportion reported feeling comfortable discussing CPF with their friends and family (25% vs 65%, respectively) and feeling a sense of community among people/families with CPF (39% vs 63%, respectively).

### CPF Diagnosis and Care

At the time of the survey, respondents reported symptoms of CPF as being present for an average of 4.5 years and obtaining a diagnosis of CPF an average of 3.5 years ago. Over half (56%) reported obtaining the diagnosis of CPF within 1 year after symptoms began and 95% within 5 years. Two respondents indicated that over a decade elapsed between CPF symptom onset and diagnosis, with 1 patient having a 32-year delay in diagnosis. Non-White respondents reported a longer mean time between symptoms and diagnosis (2.11 years) than White respondents (0.79 years). In some patients, a negative years’ difference was recorded (diagnosis before symptoms) and this was classed as zero for the purposes of data analysis. Patient gender, income, and insurance status were not associated with the timing of diagnosis reported by patients/caregivers. Most (70%) had been told that their CPF was complex, although 9% of patients/caregivers were unsure about the complexity of their CPF.

**Figure 1.**
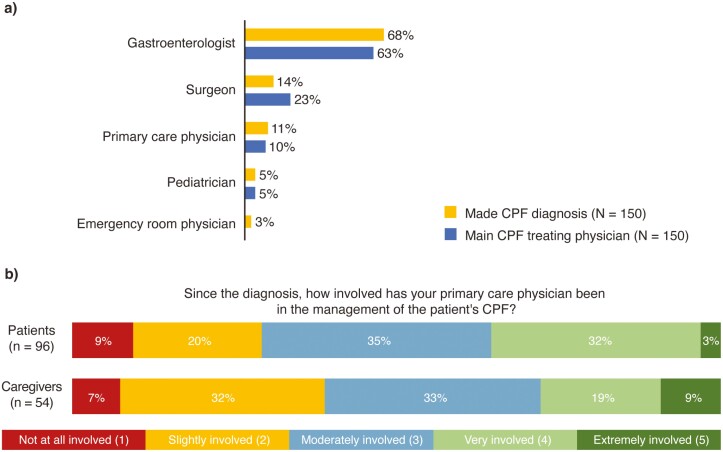
(A) Proportion of Crohn’s perianal fistula diagnoses made and primarily treated by medical specialty. (B) Patient/caregiver perception of primary care provider involvement in Crohn’s perianal fistula management after referral to a specialist. Sum of percentages may be just over or just under 100% due to rounding. CPF, Crohn’s perianal fistulas.

A GE made the diagnosis of CPF in 68% of responses (Figure 1A). Most other CPF diagnoses were made by a surgeon (14%), primary care physician (PCP; 11%), or pediatrician (5%). Caregivers were more likely to report that the diagnosis was made by a pediatrician (9% of caregivers vs 2% of patient respondents). In most cases, the type of specialist making the diagnosis was also the primary treating physician for the patient’s CPF, with 63% primarily treated by a GE. Surgeons were more likely to be the primary treating physician compared with other specialists than to make the initial diagnosis (23% vs 14%, respectively).

One-third of patients and one-third of caregivers reported that their PCP remained very or extremely involved in their CPF care following diagnosis (Figure 1B). Less than 10% of patients and caregivers (9% and 7%, respectively) indicated that their PCP did not remain involved.

### Treatment Goals and Treatments

Healing the fistula was chosen as the primary (most important) CPF goal by 39% of respondents. Decreasing pain and improving the ability to complete daily activities were chosen as the primary goal by 38% and 10% of respondents, respectively. A lower proportion of respondents chose decreasing drainage or discharge and improving the ability to maintain daily hygiene as their primary goal (9% vs 3%, respectively). For non-White respondents, 54% chose healing as the primary goal compared with 36% of White respondents, and 29% chose reducing pain as a primary goal compared with 40% of White respondents. For patients primarily managed by surgeons, a lower proportion of respondents chose fistula healing as their primary goal compared with decreasing pain (32% vs 62%, respectively). This contrasted with those managed by other physicians: 39%–53% chose fistula healing as their primary goal versus 27%–32% who chose decreasing pain as the primary goal.

Additionally, patients and caregivers varied in their primary (most important) goal of treatment. Healing the fistula was expressed as their primary goal by 43% of patients and 35% expressed reducing pain as their primary goal, compared with 33% and 43% of caregivers, respectively. A lower proportion of patients versus caregivers chose reduction in drainage as the primary goal (7% vs 13%).

Most respondents (77%) reported pharmacotherapy being received for underlying CD and CPF at the time of the survey (Table 2). Patients were taking conventional therapy (eg, aminosalicylates, antimetabolites, immunomodulators) and/or biologics. The most common current medications for the underlying CD were aminosalicylates (5-ASAs; 51%), anti-tumor necrosis factors (anti-TNFs; 50%), and calcineurin inhibitors (41%), while the most common medications for treating CPF were antimetabolites (34%), anti-TNFs (32%), and thiopurine (29%). In total, 74% of patients/caregivers reported that patients were currently taking a biologic therapy (anti-TNFs and/or other monoclonal antibody) for CD and/or CPF. A higher proportion of White respondents reported current use of biologic therapy than non-White respondents (78% vs 64%). Regarding CPF treatment, patients managed by PCPs were more likely to receive a monoclonal antibody (eg, vedolizumab, ustekinumab) other than anti-TNFs when compared with those managed by GEs or surgeons (46% vs 16% and 27%, respectively).

**Table 2.** Patient treatment for Crohn’s perianal fistulas (*N* = 150).

Current medications	Taking for Crohn’s disease,	Taking for CPF,
	*n* (%)	*n* (%)
None	20 (13%)	34 (23%)
5-aminosalicylate	77 (51%)	30 (20%)
Antimetabolite	44 (29%)	51 (34%)
Thiopurine	45 (30%)	44 (29%)
TNF inhibitor	75 (50%)	48 (32%)
Other monoclonal antibody	24 (16%)	33 (22%)
Calcineurin inhibitor	62 (41%)	32 (21%)
Other	4 (3%)	1 (1%)
Surgeries	Undergone procedure,	
	*n* (%)	
Abscess drainage	78 (52%)	
Fistula opening or widening	75 (50%)	
Seton placement for fistula drainage	47 (31%)	
Medical plug or glue	34 (23%)	
Flap procedure	24 (16%)	
Ileostomy or colostomy^a^	16 (11%)	
LIFT procedure	1 (1%)	
Bowel resection	2 (1%)	
None	21 (14%)	

^a^Temporary or permanent.

CPF, Crohn’s perianal fistulas; LIFT, ligation of intersphincteric fistula tract; TNF, tumor necrosis factor.

Patients were taking conventional therapy (eg, aminosalicylates, antimetabolites, immunomodulators) and/or biologic therapies.

Some patients may have undergone multiple surgical procedures.

Most respondents (86%) reported that patients had undergone at least 1 surgical procedure for treatment of CPF or its symptoms (Table 2). The most common procedures were abscess drainage (52%) and fistula opening or widening (50%) followed by seton placement (31%), fistula plug or glue (23%), mucosal flap procedure (16%), temporary or permanent ileostomy or colostomy (11%), ligation of the fistula tract (3%), and bowel resection (1%). Caregivers were more likely than patients to report that no surgical procedures had been performed (26% vs 7%). Of patients who had undergone surgical procedures, a lower average number of surgeries was reported for non-White respondents than White respondents (1.6 vs 2.3), and the rate of each surgery type was lower for non-White than White respondents.

### Outcomes

**Figure 2.**
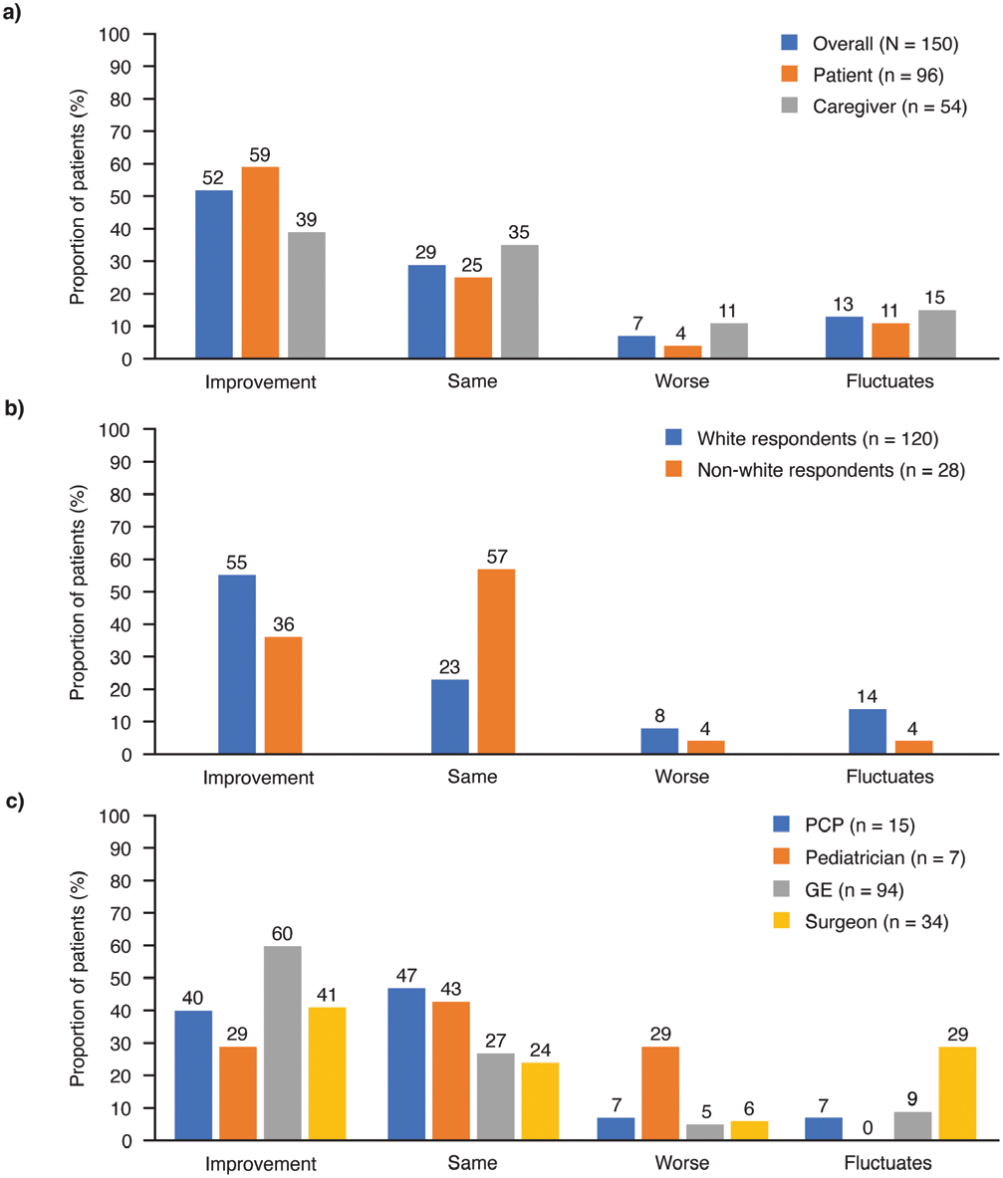
Status of Crohn’s perianal fistula symptoms since time of diagnosis (A) reported by patients versus caregivers, (B) reported by White vs non-White respondents, (C) reported by respondents for patients with different primary treating physicians (PCP, pediatrician, GE, or surgeon). Sum of percentages may be just over or just under 100% due to rounding. GE, gastroenterologist; PCP, primary care practitioner.

Overall, the majority (52%) of patients/caregivers reported that, since diagnosis, CPF symptoms had improved, while 29% reported symptoms had stayed about the same, 7% reported symptoms worsened, and 13% reported symptoms had fluctuated over time (Figure 2). Results varied when analyzed by race/ethnicity: for non-White respondents, the majority (57%) reported symptoms remained the same versus 23% of White respondents. For White respondents, 56% reported symptoms had improved compared with 36% of non-White respondents. Results also varied by patient and caregiver responses. A higher proportion of patients reported that their symptoms had improved compared with caregivers (59% vs 39%), whereas 25% of patients versus 35% of caregivers reported their symptoms had remained the same. Analyzing outcomes by primary treating physician type, of patients with reported symptom improvement, the highest proportion were primarily treated by GEs and surgeons (60% and 41%, respectively). For those treated by PCPs and pediatricians, the highest proportion of patients/caregivers reported symptoms staying the same (47% and 43%, respectively) compared with the other categories.

Most respondents were satisfied with the quality of care received (65%) and their access to care (67%) (Table 3). However, a lower proportion of non-White respondents compared with White respondents reported satisfaction with care quality (39% vs 72%, respectively) and access to care (57% vs 69%, respectively).

**Table 3.** Patient/caregiver perceptions of physicians involved in Crohn’s perianal fistula care and perceptions related to the burden of Crohn’s perianal fistula and satisfaction with care (N = 150).

	Level of agreement, *n* (%)				
Statement	Strongly disagree	Disagree	Neutral/ unsure	Agree	Strongly agree
I feel the main doctor who treats my/the patient’s CPF is knowledgeable about the disease and its treatment	1 (1%)	9 (6%)	29 (19%)	57 (38%)	54 (36%)
I know more about CPF than the doctor	30 (20%)	49 (33%)	46 (31%)	17 (11%)	8 (5%)
I am comfortable discussing fistulas with my healthcare providers	2 (1%)	5 (3%)	25 (17%)	82 (55%)	36 (24%)
I feel comfortable talking to my CPF doctor about my/the patient with CPF’s physical health	0 (0%)	12 (8%)	37 (25%)	75 (50%)	26 (17%)
I feel comfortable talking to my CPF doctor about my/the patient with CPF’s mental/emotional health	1 (1%)	14 (9%)	37 (25%)	69 (46%)	29 (19%)
I feel like I am a member of a community of people/families with CPF	9 (6%)	14 (9%)	40 (27%)	61 (41%)	26 (17%)
I am satisfied with my/the patient with CPF’s current quality of care	4 (3%)	8 (5%)	41 (27%)	73 (49%)	24 (16%)
I am satisfied with my/the patient with CPF’s current access to care	1 (1%)	•12 (8%)	37 (25%)	72 (48%)	28 (19%)
CPF disrupts the career/job of me and my family	13 (9%)	17 (11%)	27 (18%)	65 (43%)	28 (19%)
CPF creates increased stress on me and my family	3 (2%)	7 (5%)	41 (27%)	58 (39%)	41 (27%)
CPF creates increased financial burden on me and my family	2 (1%)	12 (8%)	46 (31%)	63 (42%)	27 (18%)
CPF creates increased emotional burden on me and my family	1 (1%)	14 (9%)	39 (26%)	59 (39%)	37 (25%)

CPF, Crohn’s perianal fistulas.

### Decision-Making and Healthcare Professional Interactions

**Figure 3.**
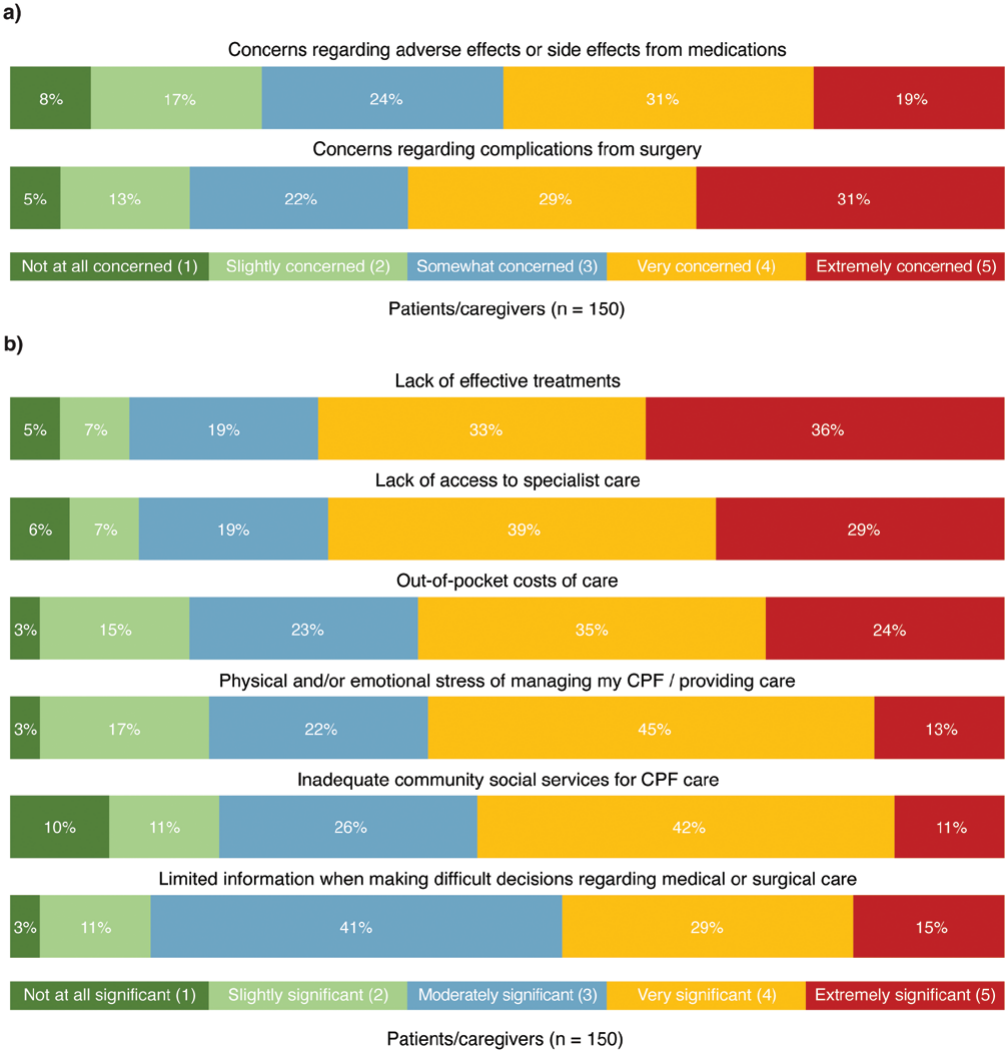
(A) Patient/caregiver level of concern of adverse effects from medications and complications from surgery. (B) Patient/caregiver perceived significance of barriers to the optimal care of Crohn’s perianal fistulas.

Most respondents expressed comfort in discussing CPF (79%), physical health (67%), and mental health (65%) with their healthcare providers, regardless of physician type. A greater proportion of patients/caregivers reported very/extreme concern regarding complications of surgery (60%) than side effects of medications (50%) (Figure 3A). The main concerns surrounding surgery for CPF were related to its success rate, safety, recovery process, risk of recurrence, and complications leading to organ damage. Patients with a low level of concern related to surgery expressed an interest in long-term disease control and the well-established nature of CPF procedures.

Nearly all patients/caregivers wanted to be involved in treatment decision-making with their physicians (81%). Some preferred to make the final decision about their treatment after considering the doctor’s opinion (41%). Some preferred to share responsibility equally with the doctor for deciding which treatments are best (29%) or have the doctor make the final decision after considering their opinion (14%). Few patients preferred to make decisions themselves (11%) or leave all decision-making to the doctor (5%). Most patients/caregivers who reported that the physician made the final treatment decision preferred to continue to let the doctor make those decisions (75%). Those who already shared decision-making with the HCP would like to continue with this model (60%) or would like to have more control over the decision (38%), while few wanted the HCP to make the final decision after considering their opinion (2%). For patients/caregivers who already make the final decision, most would like to continue with their model (79%), while 19% would like to share decision-making with their physicians.

Overall, patients/caregivers indicated a level of confidence in their treating physicians, with 74% reporting that they felt the main treating physician was knowledgeable about CPF. Of concern is that 16% perceived that they knew more about CPF than their physician (Table 3). For patients treated by GEs and surgeons, the majority of patients/caregivers were confident that their treating physician was knowledgeable about CPF (77% and 85%, respectively). This was in contrast to patients managed primarily by a PCP or pediatrician in which a smaller proportion of patients/caregivers were confident that their physician was knowledgeable about CPF (53% and 29%, respectively). For patients treated by GEs, surgeons, and pediatricians, a lower proportion of patients/caregivers felt they knew more about CPF than their physician (11%, 21%, and 0% respectively) compared with patients treated by a PCP (53%).

### Barriers to CPF Treatment

Patients/caregivers perceived several barriers to receiving the optimal CPF care (Figure 3B). Over half reported that lack of effective treatments (69%) and lack of access to specialist care (68%) were very or extremely significant barriers. Other very/extremely significant barriers included out-of-pocket costs of care (59%), stress of managing CPF (58%), inadequate community social services for CPF care (53%), and limited information to make treatment decisions (45%). Non-White and White respondents had similar perceptions on the barriers of treatment options and access to care (68–70% reported them as very/extremely significant), in contrast to the other barriers (out-of-pocket costs, stress, inadequate community social services, and limited information) which were reported as very/extremely significant by a lower proportion of non-White respondents (39–43%) compared with White respondents (46–64%).

Sources of CPF information considered very valuable to patients/caregivers were GEs (51%), surgeons (33%), patient advocacy organizations websites (27%), pharmacists (21%), and other patients with CPF (9%) (Table 4). Only 50% of non-White respondents versus 69% of White respondents agreed or strongly agreed that they knew where to go to get CPF information. Most patients/caregivers (69%) stated that they would benefit from more information on managing day-to-day CPF symptoms, and this was higher for White than non-White respondents (74% vs 50%).

**Table 4.** Use and perception of value of Crohn’s perianal fistula information sources by patients/caregivers and informal needs (*N* = 150).

Information source	Never used, *n* (%)	Used, not valuable, *n* (%)	Used, somewhat valuable, n (%)	Used, very valuable, *n* (%)	
Gastroenterologists	7 (5%)	20 (13%)	46 (31%)	77 (51%)	
Surgeons	11 (7%)	18 (12%)	71 (47%)	50 (33%)	
Patient advocacy organization websites (eg, Crohn’s & Colitis Foundation)	23 (15%)	31 (21%)	55 (37%)	41 (27%)	
Pharmacists	20 (13%)	34 (23%)	64 (43%)	32 (21%)	
Other patients are parents/caregivers with CPF	21 (14%)	33 (22%)	82 (55%)	14 (9%)	
Drug company websites (companies that provide CPF medications and treatments)	25 (17%)	35 (23%)	69 (46%)	21 (14%)	
General social media websites (eg, Twitter, Facebook)	17 (11%)	48 (32%)	70 (47%)	15 (10%)	
Family members and/or caregivers	16 (11%)	55 (37%)	48 (32%)	31 (21%)	
Primary care physicians	15 (10%)	55 (37%)	62 (41%)	18 (12%)	
General health education websites (eg, WebMD, Mayo Clinic)	14 (9%)	58 (39%)	69 (46%)	9 (6%)	
Friends	28 (19%)	60 (40%)	52 (35%)	10 (7%)	
Books or magazines from advocacy groups	33 (22%)	57 (38%)	51 (34%)	9 (6%)	

	Level of agreement, *n* (%)				
Statement	Strongly disagree	Disagree	Neutral/ unsure	Agree	Strongly agree
I would benefit from more information about CPF clinical trials	2 (1%)	8 (5%)	39 (26%)	68 (45%)	33 (22%)
I would benefit from more information about managing day-to-day CPF symptoms	0 (0%)	11 (7%)	35 (23%)	74 (49%)	30 (20%)
I know where to go if I want to get information about CPF	2 (1%)	14 (9%)	36 (24%)	74 (49%)	24 (16%)
I would benefit from more interaction with other people and/or families/caregivers affected by CPF	5 (3%)	17 (11%)	40 (27%)	71 (47%)	17 (11%)
I have adequate resources online to answer my questions on CPF	3 (2%)	28 (19%)	40 (27%)	62 (41%)	17 (11%)

CPF, Crohn’s perianal fistulas.

## Discussion

This national survey of patients and their caregivers highlights their perceptions and needs related to CPF management. Despite the introduction of new CD pharmacotherapies and evolution of CPF surgical procedures, patients continue to experience significant burdens related to CPF. The survey also identified areas of disparities in care among patient groups and potential areas in which treatment outcomes may be improved.

Patients and caregivers noted that the time to diagnosis could range from less than 1 year to 32 years, with non-White patients having more than double the mean time from symptoms to diagnosis than White patients. It was also identified that CPF diagnosis primarily came from GEs, and almost all patients/caregivers reported that their PCPs remained involved in CPF care. However, patients primarily treated by a PCP were more likely to feel they (or their caregiver) knew more about CPF than their physician compared with patients treated by other physicians.

Our results indicate that patients and caregivers had an overall primary CPF treatment goal of fistula healing, followed closely by pain reduction. However, the primary goal differed by race/ethnicity and also by primary treating physician type. In conducting a parallel survey of physicians who manage CPF (GEs and surgeons), we found that GEs prioritize fistula healing over improving quality of life or resolving fistula symptoms, while the primary goal of surgeons was improving quality of life (unpublished data), which may be a reflection of the current choices of surgery available.

The current survey identified that, while most respondents had confidence in their treating physician, a gap was identified as 16% reported they knew more about CPF than their doctors. There is a need to ensure that all physicians are aware of CPF and its optimal management.

Overall, 65% of respondents were satisfied with their quality of care, although satisfaction was lower in non-White respondents than in White respondents. This corresponds with 52% of patients/caregivers reporting symptom improvement, but results varied by race/ethnicity and by treating physician type. The survey identified the need for optimizing treatment outcomes for patients.

While 74% of patients were currently taking biologics for their CD and/or CPF, the rates varied by race/ethnicity with non-White respondents reporting lower rates of biologic use than White respondents. This may reflect a need for optimization of medical therapy and access to medicines due to insurance coverage and/or out-of-pocket costs. Additionally, while most patients had undergone a surgical procedure, setons were only used in one-third of patients. This may reflect the level of individual disease characteristics, or the choice of surgical procedure may reflect that treatment algorithms specific to CPF may not be well understood by the greater surgical community.

In our parallel survey of clinicians who manage CPF (GEs, surgeons, and their associated nurse practitioners and physician assistants), we found 45% considered patient reluctance to undergo recommended treatment as a very significant or extremely significant barrier to optimal management (unpublished data). Considering this in the context of results from our patient survey, a lack of understanding of surgical treatment options may contribute to or cause this perceived reluctance.

CPF requires multidisciplinary management by a variety of HCPs. Our survey found that a greater proportion of patients primarily treated by GEs reported symptom improvement compared with surgeons, PCPs, or pediatricians. This may be reflected in the choice of medicines prescribed by GEs versus other providers. Additionally, a lack of access to specialist care was felt to be one of the most significant barriers to receiving optimal CPF care. The survey did not evaluate the referral pathway over the course of patients’ disease, although it did identify the type of HCP who diagnosed and/or primarily managed patients’ CPF. It could be that insurance providers may limit easy access to specialists and obtaining access to care from these specialists as quickly as possible may be reflected by the responses. Further education on optimal CPF multidisciplinary team operations may improve access.

The survey identified that most patients/caregivers wanted to be involved in the decision-making process for their CPF. In our parallel survey of physicians who treat CPF, 47% of GEs versus 51% of surgeons wanted patients to share treatment decision-making with them (unpublished data). HCPs should involve patients consistently in the treatment decision-making process as research has shown that patient treatment preferences in CD do not align with that of clinicians.^18^ Furthermore, a patient-centered multidisciplinary approach is required as colorectal surgeon treatment preferences have been reported to be significantly different to those of GEs.^18^

An estimated 71%–84% of CPF worldwide meet the American Gastroenterological Association’s definition of a “complex” fistula, which is in alignment with our overall results.^19^ Nonetheless, we identified an uncertainty about fistula complexity among respondents and a lower proportion of caregivers compared with patients who reported complex disease. Some patients and caregivers may not be adequately informed about the complex nature of their or their family member’s CPF, which could negatively impact their ability to participate in treatment decisions and understand surgical risks.

Having limited information for treatment decision-making was reported as a significant barrier by a lower proportion of patients/caregivers than other barriers to optimal CPF care, although some considered it to be significant. Increased patient knowledge about their condition is likely to improve both clinical outcomes and perceptions of care. Moreover, stigma related to inflammatory bowel disease is associated with poor patient outcomes and increased patient knowledge about CPF could play a key role in stigma reduction.^20^

The survey findings suggest that there is a need for better patient information related to medical treatments for CPF. Although most patients were on medication for their CPF, and respondents indicated somewhat less concern about medication adverse effects compared with complications of surgery, 92% reported having some level of concern about medications. Specific concerns mentioned by patients/caregivers covered a broad range of effects, ranging from specific side effects to overall functioning. With no clear guidelines on medication sequencing in CPF and an increasing number of pharmacotherapies available for treatment, patients may benefit from information on medical treatment options and associated risks. Further, physician education that assists PCPs and specialists in discussing medical treatment options may assist patients in playing a role in their treatment decisions.

Specific concerns related to information on CPF that arose in qualitative data included the need for information that is personalized and is specific to patients with co-existing CD. An informational topic of particular relevance is post-surgical care. One survey respondent who indicated a desire for post-surgical tips and instructions wrote that “much of the information about perianal fistulas on the Internet is geared toward people without underlying [inflammatory bowel disease], even the discharge notes given by my surgeon were not catered toward people with CD and contained instructions that would be pretty detrimental to my management of Crohn’s.” This observation aligns with data from other studies. An investigation of the informational preferences of patients who have undergone surgery for CPF identified dissatisfaction with the information given on nontechnical features of procedures, such as aftercare and activities of daily living.^21^ Another survey-based study identified wound and aftercare, effect on perianal symptoms, and severity of surgery (risks of procedure, procedure-related pain, and invasiveness) as information needs of patients undergoing surgery for CPF.^22^ Participants in a qualitative exploration of patient CPF experiences reported that physicians often did not explain goals and methods of treatment coherently or provide realistic expectations about setons and pain following surgery.^17^

The emphasis on a need for surgical-related information may reflect the primary management of CPF by a GE, rather than a surgeon. It is possible that surgeons assume the patient’s GE will provide education regarding perioperative care. A lack of detailed, personalized education about surgery may also reflect surgeons’ time constraints or a lack of availability of relevant information. Research indicates that postoperative patients with CPF turn to other sources for information, with 1 study revealing they access online health information—particularly patient forums—to find out more about life after surgery.^23^

Identifying ways to educate patients and their HCPs on CPF may help with recognition of CPF and result in earlier diagnosis and improve the patient journey. Continuing education about CPF diagnosis and management for all HCPs who have patients with CPF may raise awareness of the differential diagnosis, treatment options, and referral to specialists, which may improve treatment outcomes.

### Study Limitations

Few caregivers of pediatric patients were included, and the referral between pediatricians and pediatric GEs was not discerned, nor was the transfer of pediatric patients to adult care. This is an area for future research. The HCP referral pathway was also not investigated.

The study design limited our results to subjective data. CPF diagnosis was self-reported by survey respondents, and we did not attempt to verify patient responses through medical record review or any other means. A portion of respondents were recruited by physicians who treat CD and CPF. As a result, our data may overrepresent patients who are actively seeing 1 or more medical specialists for their CPF care and underrepresent those who are not currently undergoing treatment for their CPF or who have not recently sought medical consultation for CPF. Responder and recall biases may affect the qualitative aspects of our study. It is possible that respondents with strongly positive or negative feelings toward a question were more likely to offer written insight. Differences observed between respondent groups were observed differences and may not be statistically significant.

## Conclusions

This study provides patient-centered information that can inform physicians’ approaches to CPF management. CPF places physical, emotional, and financial burdens on patients and caregivers, and barriers to care are common. Patients/caregivers prefer to be involved in management decisions yet perceive the need for additional information to do so.

Racial disparities in diagnosis and management of CPF exist, including length of time to diagnosis, perceptions of quality of care, and barriers to treatment. Differences in treatment approach and perceptions of care also vary by the type of provider who is directing care. Further research into inconsistencies in the management of CPF is needed, as is CPF information for patients/caregivers and healthcare provider education on managing CPF in various populations.

We expect that the results of this study will assist medical education providers in considering key aspects related to CPF diagnosis and treatment that physicians may benefit from, particularly with regard to incorporating the patient perspective into decision-making, patient-directed education, integrating quality of life aspects, and coordination of care.

## Supplementary Material

otad081_suppl_Supplementary_Data

## Data Availability

The datasets used and/or analyzed during the current study are available from the corresponding author on reasonable request.
