# Supplementary material for: Patient and Caregiver Perspectives on Their Experiences With Crohn’s Perianal Fistulas
Source: Crohns Colitis 360. 2024 Jan 6;6(1):otad081. doi: 10.1093/crocol/otad081 (PMC10803099; doi:10.1093/crocol/otad081)
Supplement: otad081_suppl_Supplementary_Data [file otad081_suppl_supplementary_data.docx]

**Supplementary Information 1**

1. **What is your age? _______**

(If less than 21 years old: *“Thank you for your interest in our survey. However, you do not currently meet the criteria for this study.”)*

1. **What is your relationship to a person with Crohn’s disease who also has a perianal fistula (CPF)?** (select only one)

| ❑ | I have a CPF |
| --- | --- |
| ❑ | I am a parent of an individual with CPF |
| ❑ | I am a family caregiver (nonparent) of an individual with CPF |
| ❑ | I have no relationship to an individual with Crohn’s disease who has CPF |

<If no relationship is selected above: *“Thank you for your interest in our survey. However, you do not currently meet the criteria for this study.”>*

<If the person completing the survey is identified as having perianal fistulas – go to patient survey. If “parent/caregiver” – go to caregiver survey. Survey differences are highlighted in yellow>

**2a. [If “caregiver” in Q2] How old is the person with CPF?** **_______**

1. **Have you/you or the person with PF ever been told by a healthcare provider that it was “complex”?** (select only one)

| ❑ | Yes |
| --- | --- |
| ❑ | No |
| ❑ | Unsure |

1. **Approximately how many years ago did you/the person with CPF first start having symptoms you now associate with CPF?** (If within the last year, please enter “0” years) _____________
2. **Approximately how many years ago were you/was the person formally diagnosed with CPF?** (If within the last year, please enter “0” years) _____________
3. **What type of physician made the CPF diagnosis?** (select only one)

| ❑ | Primary care physician (ie, family physician, general internist) |
| --- | --- |
| ❑ | Pediatrician |
| ❑ | Gastroenterologist |
| ❑ | Surgeon |
| ❑ | Emergency room physician |
| ❑ | Other (please specify) |

1. **What type of doctor is the main treater of your/the person’s CPF?** (select only one)

| ❑ | Primary care physician (ie, family physician, general internist) |
| --- | --- |
| ❑ | Pediatrician |
| ❑ | Gastroenterologist |
| ❑ | Surgeon |
| ❑ | Other (please specify) |

1. **Since your diagnosis, how involved has your primary care physician been in the management of your CPF?**

| Not at all involved | Slightly involved | Somewhat involved | Very involved | Extremely involved |
| --- | --- | --- | --- | --- |
| ❑ | ❑ | ❑ | ❑ | ❑ |

1. **Have you/the person with CPF had any of the following surgeries related to fistula care?** (select all that apply)

| ❑ | Seton (a thread or cord) placement to drain the fistula |
| --- | --- |
| ❑ | Abscess drainage |
| ❑ | Medical plug or glue |
| ❑ | Fistula opening or widening |
| ❑ | Flap procedure (advancement flap or mucosal flap) |
| ❑ | Ileostomy or colostomy, temporary or permanent |
| ❑ | Other (please specify) |
| ❑ | I have/The person with CPF has not had any surgeries related to fistulas <make answer option exclusive> |

1. **What medicines are you/the person with CPF taking now for Crohn’s or the PF?** (select all that apply)

| Crohn’s | CPF |  |
| --- | --- | --- |
| ❑ | ❑ | 5-aminosalicylate (eg, sulfasalazine, mesalamine, Azulfidine, Canasa, Delzicol) |
| ❑ | ❑ | Anti-metabolite (eg, methotrexate, Otrexup, Xatmep, Trexall) |
| ❑ | ❑ | Anti-TNF agent (eg, infliximab, adalimumab, Certolizumab, Cimzia, Humira, Remicade) |
| ❑ | ❑ | Calcineurin inhibitor (eg, tacrolimus, cyclosporin, Envarsus, Protopic, Sandimmune, Neoral, Gengraf) |
| ❑ | ❑ | Other monoclonal antibody (eg, vedolizumab, ustekinumab, Entyvio, Stelara) |
| ❑ | ❑ | Thiopurine (eg, azathioprine, 6-mercaptopurine, Azasan, Purixan) |
| ❑ | ❑ | Other (please specify) |
| ❑ | ❑ | I am/The person with CPF is not taking any treatment for CPF <make answer option exclusive> |

1. **What medicines have you/the person with CPF ever taken in the past?** (select all that apply)

| Crohn’s | CPF |  |
| --- | --- | --- |
| ❑ | ❑ | 5-aminosalicylate (eg, sulfasalazine, mesalamine, Azulfidine, Canasa, Delzicol) |
| ❑ | ❑ | Anti-metabolite (eg, methotrexate, Otrexup, Xatmep, Trexall) |
| ❑ | ❑ | Anti-TNF agent (eg, infliximab, adalimumab, Certolizumab, Cimzia, Humira, Remicade) |
| ❑ | ❑ | Calcineurin inhibitor (eg, tacrolimus, cyclosporin, Envarsus, Protopic, Sandimmune, Neoral, Gengraf) |
| ❑ | ❑ | Other monoclonal antibody (eg, vedolizumab, ustekinumab, Entyvio, Stelara) |
| ❑ | ❑ | Thiopurine (eg, azathioprine, 6-mercaptopurine, Azasan, Purixan) |
| ❑ | ❑ | Other (please specify) |
| ❑ | ❑ | I am/The person with CPF is not taking any treatment for CPF <make answer option exclusive> |

1. **How concerned are you about potentially having each of the following as a result of CPF treatment?**

| (select one number for each) | Not at all concerned | Slightly concerned | Somewhat concerned | Very concerned | Extremely concerned |
| --- | --- | --- | --- | --- | --- |
| Adverse effects or side effects from medications | 1 | 2 | 3 | 4 | 5 |
| Complications from surgery | 1 | 2 | 3 | 4 | 5 |

**12a. [If indicated 2-5 on adverse effects from medication] What are your concerns regarding medication side effects? _____________**

**12b. [If indicated 2-5 on complications from surgery] What are your concerns regarding surgery? _____________**

1. **Please rank each of the following goals related to CPF treatment, where 1 is the most important goal to achieve and 5 is the least important goal.**

Healing the fistula __

Decreasing pain __

Decreasing drainage or discharge __

Improving the ability to complete daily activities __

Improving the ability to maintain hygiene __

1. **Since my/the patient with CPF’s diagnosis…** (select only one)

|  | Symptoms have improved |
| --- | --- |
|  | Symptoms have stayed about the same |
|  | Symptoms have worsened |
|  | Symptoms have fluctuated over time |

1. **Please rate your level of agreement with the following:**

| (select one number for each) | Strongly disagree | Disagree | Neutral/Unsure | Agree | Strongly agree |
| --- | --- | --- | --- | --- | --- |
| It is important to have multiple doctors from different specialties involved in the management of my CPF | 1 | 2 | 3 | 4 | 5 |
| Fistulas in people with Crohn disease need to be treated differently than fistulas in people who don’t have Crohn disease | 1 | 2 | 3 | 4 | 5 |
| Once a fistula heals, there is a high chance that it will recur | 1 | 2 | 3 | 4 | 5 |

1. **Which approach best characterizes how a final decision is generally made regarding your/the person with CPF’s management?** (select only one)

|  | I/We make the final decision about which treatments I/the person with CPF receive/s |
| --- | --- |
|  | I/We make the final decision about my/the person with CPF’s treatments after seriously considering the doctor’s opinion |
|  | The doctor and I/The doctor, the person with CPF, and I share responsibility for deciding which treatments are best |
|  | The doctor makes the final decision about which treatments are used, but seriously considers my/our opinion |
|  | I/We leave all decisions regarding my/the person with CPF’s treatments to the doctor |

1. **Which approach best characterizes how you prefer a final decision is made regarding your/the person’s future CPF management?** (select only one)

|  | I/We prefer to make the final decision about which treatments I/the person with CPF will receive |
| --- | --- |
|  | I/We prefer to make the final decision about my/the person with CPF’s treatments after seriously considering my/the doctor’s opinion |
|  | I/We prefer that my doctor and I/the doctor, the person with CPF, and I share responsibility for deciding which treatments are best |
|  | I/We prefer that my doctor make the final decision about which treatments will be used, but seriously consider my/our opinion |
|  | I/We prefer to leave all decisions regarding my/the person with CPF’s treatments to the doctor |

1. **Please rate your level of agreement with the following:**

| (select one number for each) | Strongly disagree | Disagree | Neutral | Agree | Strongly agree |
| --- | --- | --- | --- | --- | --- |
| I feel like I am a member of a community of people/families with CPF | 1 | 2 | 3 | 4 | 5 |
| I am satisfied with my/the person with CPF’s current quality of care | 1 | 2 | 3 | 4 | 5 |
| I am satisfied with my/the person with CPF’s current access to care | 1 | 2 | 3 | 4 | 5 |
| CPF disrupts the career/job of me and my family | 1 | 2 | 3 | 4 | 5 |
| CPF creates increased stress on me and my family | 1 | 2 | 3 | 4 | 5 |
| CPF creates increased financial burden on me and my family | 1 | 2 | 3 | 4 | 5 |
| CPF creates increased emotional burden on me and my family |  |  |  |  |  |

1. **Please rate your level of agreement with the following:**

| (select one number for each) | Strongly disagree | Disagree | Neutral | Agree | Strongly agree |
| --- | --- | --- | --- | --- | --- |
| I am comfortable discussing fistulas with those I am close to (such as friends or family members) | 1 | 2 | 3 | 4 | 5 |
| I am comfortable discussing fistulas with my healthcare providers | 1 | 2 | 3 | 4 | 5 |

1. **How significant are each of the following problems or barriers to receiving the best possible CPF care?**

| (select one number for each) | Not significant | Slightly significant | Somewhat significant | Very significant | Extremely significant |
| --- | --- | --- | --- | --- | --- |
| Inadequate community social services for CPF care | 1 | 2 | 3 | 4 | 5 |
| Out-of-pocket costs of care | 1 | 2 | 3 | 4 | 5 |
| Limited information when making difficult decisions regarding medical or surgical care | 1 | 2 | 3 | 4 | 5 |
| Physical and/or emotional stress of managing my CPF / providing care | 1 | 2 | 3 | 4 | 5 |
| Lack of access to specialist care | 1 | 2 | 3 | 4 | 5 |
| Lack of effective treatments | 1 | 2 | 3 | 4 | 5 |

1. **What other problems have you/has the person with CPF experienced in obtaining the best possible care for your/their CPF?** (please describe)
2. **Have you/you or the person with CPF received financial support from an organization, such as a pharmaceutical company or advocacy group, to help pay for CPF care or medication**? (select only one)

| ❑ | Yes |
| --- | --- |
| ❑ | No |
| ❑ | Unsure |

1. **Please rate your level of agreement with the following:**

| (select one number for each) | Strongly disagree | Disagree | Neutral | Agree | Strongly agree |
| --- | --- | --- | --- | --- | --- |
| I feel the main doctor who treats my/the person’s CPF is knowledgeable about the disease and its treatment | 1 | 2 | 3 | 4 | 5 |
| I know more about CPF than the doctor | 1 | 2 | 3 | 4 | 5 |
| I feel comfortable talking to my CPF doctor about my/the person with CPF’s physical health | 1 | 2 | 3 | 4 | 5 |
| I feel comfortable talking to my CPF doctor about my/the person with CPF’s mental/emotional health | 1 | 2 | 3 | 4 | 5 |

1. **In general, have you previously heard of stem cell therapy?** Yes/no <If yes display parts a b c and dc>

**a.** **Have you heard of stem cell therapy being used to treat CPF?** Yes/No

**b.** **In general, what are your attitudes toward the use of stem cell therapy?**

| Strongly negative | Slightly negative | Unsure/Neutral | Slightly positive | Strongly positive |
| --- | --- | --- | --- | --- |
| ❑ | ❑ | ❑ | ❑ | ❑ |

**c. What are your attitudes toward the use of stem cell therapy for treatment of CPF?**

| Strongly negative | Slightly negative | Unsure/Neutral | Slightly positive | Strongly positive |
| --- | --- | --- | --- | --- |
| ❑ | ❑ | ❑ | ❑ | ❑ |

**d. If your healthcare providers were to suggest a stem cell treatment for management of your CPF, how concerned would you be?**

| Not at all concerned | Slightly concenred | Somewhat concerned | Very concerned | Extremely concerned |
| --- | --- | --- | --- | --- |
| ❑ | ❑ | ❑ | ❑ | ❑ |

1. **Please rate your perceptions of the following sources for information on CPF.** (select one for each)

|  | Never used | Used, not valuable | Used, somewhat valuable | Used,  very valuable |
| --- | --- | --- | --- | --- |
| Primary care physicians | ❑ | ❑ | ❑ | ❑ |
| Gastroenterologists | ❑ | ❑ | ❑ | ❑ |
| Surgeons | ❑ | ❑ | ❑ | ❑ |
| Pharmacists | ❑ | ❑ | ❑ | ❑ |
| Patient advocacy organization websites (examples: Crohn's & Colitis Foundation, etc.) | ❑ | ❑ | ❑ | ❑ |
| Books or magazines from advocacy groups | ❑ | ❑ | ❑ | ❑ |
| Other patients or parents/caregivers with CPF | ❑ | ❑ | ❑ | ❑ |
| General health education websites (examples: WebMD, Mayo Clinic) | ❑ | ❑ | ❑ | ❑ |
| Drug company websites (companies that provide CPF medications and other treatments) | ❑ | ❑ | ❑ | ❑ |
| General social media websites (examples: Twitter, Facebook) | ❑ | ❑ | ❑ | ❑ |
| Friends | ❑ | ❑ | ❑ | ❑ |
| Family members and/or caregivers | ❑ | ❑ | ❑ | ❑ |

1. **Are there any other sources of information you/you or the person with CPF have found valuable?** (please describe)
2. **Please rate your level of agreement with the following:**

| (select one number for each) | Strongly disagree | Disagree | Neutral | Agree | Strongly agree |
| --- | --- | --- | --- | --- | --- |
| I know where to go if I want to get information about CPF | 1 | 2 | 3 | 4 | 5 |
| I have adequate resources online to answer my questions on CPF | 1 | 2 | 3 | 4 | 5 |
| I would benefit from more interaction with other people and/or families/caregivers affected by CPF | 1 | 2 | 3 | 4 | 5 |
| I would benefit from more information about managing day-to-day CPF symptoms | 1 | 2 | 3 | 4 | 5 |
| I would benefit from more information about CPF clinical trials | 1 | 2 | 3 | 4 | 5 |
| I am interested in learning more about stem cell therapy for CPF treatment | 1 | 2 | 3 | 4 | 5 |

1. **What types of educational materials about CPF would you like more of?** (select all that apply)

|  | Paper pamphlets or handouts |
| --- | --- |
|  | Information about tablet/smartphone apps |
|  | Link to a specific website |
|  | Information about patient support groups |
|  | Information about CPF organizations/advocacy groups |
|  | Other (please specify) |
|  | None of these <make answer option exclusive> |

1. **What one informational or educational topic related to CPF would be most valuable to you/you or the person with CPF?** (please describe)
2. **How would you describe your background?** (select all that apply)

| ❑ | American Indian or Alaska Native |
| --- | --- |
| ❑ | Asian |
| ❑ | Black/African American |
| ❑ | Hispanic/Latino |
| ❑ | Native Hawaiian or Other Pacific Islander |
| ❑ | White, non-Hispanic |
| ❑ | Other ­­(please specify) |
| ❑ | Prefer not to say |

1. **What of the following best describes your gender?** (select only one)

| ❑ | Male |
| --- | --- |
| ❑ | Female |
| ❑ | Non-binary |
| ❑ | Prefer not to say |
| ❑ | Prefer to self-describe __________________ |

1. **What is your current work status?** (select only one)

| ❑ | Employed for wages – full-time |
| --- | --- |
| ❑ | Employed for wages – part-time |
| ❑ | Self-employed |
| ❑ | Out of work and looking for work |
| ❑ | Out of work but not currently looking for work |
| ❑ | A homemaker |
| ❑ | A student |
| ❑ | Military |
| ❑ | Retired |
| ❑ | Unable to work |

1. **What is your total household income?** (select only one)

| ❑ | Less than $10,000 |
| --- | --- |
| ❑ | $10,000 to $29,999 |
| ❑ | $30,000 to $49,999 |
| ❑ | $50,000 to $69,999 |
| ❑ | $70,000 to $89,999 |
| ❑ | $90,000 to $119,999 |
| ❑ | $120,000 to $149,999 |
| ❑ | $150,000 or more |

1. **What is the highest degree or level of education you have completed?** (select only one)

| ❑ | Some high school |
| --- | --- |
| ❑ | High school graduate (includes equivalency) |
| ❑ | Trade/technical/vocational training |
| ❑ | Some college, no degree |
| ❑ | Associate’s degree |
| ❑ | Bachelor’s degree |
| ❑ | Master’s degree |
| ❑ | Doctoral degree (PhD) |
| ❑ | Professional degree (MD, JD, etc) |

1. **Do you/Does the person with CPF currently have health insurance?** (select only one)

| ❑ | Yes, private insurance |
| --- | --- |
| ❑ | Yes, Medicare/Medicaid |
| ❑ | No |

1. **Please enter the 5-digit ZIP code (example, 35211) of your primary residence. ____________**
